# 3D Printed Masks for Powders and Viruses Safety Protection Using Food Grade Polymers: Empirical Tests

**DOI:** 10.3390/polym13040617

**Published:** 2021-02-18

**Authors:** Ruben Foresti, Benedetta Ghezzi, Matteo Vettori, Lorenzo Bergonzi, Silvia Attolino, Stefano Rossi, Giuseppe Tarabella, Davide Vurro, Didier von Zeppelin, Salvatore Iannotta, Andrea Zappettini, Guido Maria Macaluso, Michele Miragoli, Marcello Giuseppe Maggio, Cosimo Costantino, Stefano Selleri, Claudio Macaluso

**Affiliations:** 1 Department of Medicine and Surgery, University of Parma, 43126 Parma, Italy; benedetta.ghezzi@unipr.it (B.G.); stefano.rossi@unipr.it (S.R.); guidomaria.macaluso@unipr.it (G.M.M.); michele.miragoli@unipr.it (M.M.); marcellogiuseppe.maggio@unipr.it (M.G.M.); cosimo.costantino@unipr.it (C.C.); cmacalus@gmail.com (C.M.); 2 Center of Dental Medicine, University of Parma, 43126 Parma, Italy; 3 MaCh3D SRL, 43121 Parma, Italy; m.vettori@mach3d.it (M.V.); l.bergonzi@mach3d.it (L.B.); 4 Department of Philology, Literature and Linguistics, University of Pisa, 56125 Pisa, Italy; s.attolino@studenti.unipi.it; 5 Camlin Italy SRL, 43124 Parma, Italy; g.tarabella@camlintechnologies.com; 6 IMEM-CNR Institute, 43124 Parma, Italy; davide.vurro@imem.cnr.it (D.V.); salvatore.iannotta@imem.cnr.it (S.I.); andrea.zappettini@imem.cnr.it (A.Z.); 7 ARBURG GmbH, 72290 Loßburg, Germany; didier_von_zeppelin@arburg.com; 8 Humanitas Clinical and Research Center—IRCCS, 20089 Rozzano, Italy; 9 CERT, Centre of Excellence for Toxicology Research, University of Parma, 43126 Parma, Italy; 10 Department of Engineering and Architecture, University of Parma, 43125 Parma, Italy; stefano.selleri@unipr.it

**Keywords:** COVID-19, SARS-COV-2, 3D printing, fused deposition modeling (FDM), precision extrusion deposition (PED), electrospinning, safety protection devices (SPD), medical devices

## Abstract

The production of 3D printed safety protection devices (SPD) requires particular attention to the material selection and to the evaluation of mechanical resistance, biological safety and surface roughness related to the accumulation of bacteria and viruses. We explored the possibility to adopt additive manufacturing technologies for the production of respirator masks, responding to the sudden demand of SPDs caused by the emergency scenario of the pandemic spread of SARS-COV-2. In this study, we developed different prototypes of masks, exclusively applying basic additive manufacturing technologies like fused deposition modeling (FDM) and droplet-based precision extrusion deposition (db-PED) to common food packaging materials. We analyzed the resulting mechanical characteristics, biological safety (cell adhesion and viability), surface roughness and resistance to dissolution, before and after the cleaning and disinfection phases. We showed that masks 3D printed with home-grade printing equipment have similar performances compared to the industrial-grade ones, and furthermore we obtained a perfect face fit by customizing their shape. Finally, we developed novel approaches to the additive manufacturing post-processing phases essential to assure human safety in the production of 3D printed custom medical devices.

## 1. Introduction

During recent decades, there has been a growing interest in the use of additive manufacturing technology (AM) in biopolymers/bio-composite manufacturing, biological and medical application [1,2,3,4,5,6,7,8], demonstrating the scalability of this technology in our future daily use [9,10,11].

AM reduces waste generated from typical mass production [12,13], improves the satisfaction in subjective/individual necessity [14,15,16] of personalization, enabling the production “at home” of “everything” we need [17,18,19], in line with the autonomous smart city concept [9] and the consequent ability to manage critical environmental events.

The phases of the worldwide spreading of SARS-COV-2 require a huge amount of safety protection devices (SPD) like masks, because of the collapse of the global supply-chain of SPDs, and 3D printing should solve this necessity [20]. However, the production of these devices must respect the local Government Guideline, assuring protection against the virus, healthiness, customer safety [21,22], ergonomics, reuse (when possible), and proper air filtering, in order to prevent skin inflammation, in line with the regulation for specific use [23].

It is well known that one of the challenges in 3D printing medical application scalability is the use of toxic materials and surface roughness, which allows the deposition and proliferation of bacteria and viruses. 3D printed material requires cleaning [24] planned overtime, surface customization [25,26,27], or expensive post-processing, not always applicable to complex geometries obtained with AM.

Then, in order to assure the additive manufacturing global scalability, it is mandatory to identify materials, available in both developed and developing countries like those used for food packaging that are typically non-toxic, and to take into consideration occupied space (machine volumes, manufacturing platform), waste reduction and power requirement.

In this study, we designed and developed different printed models of masks and filter supports via material extrusion AM, evaluating the real abilities and limits in the medical device development. In particular, we used for the production of frame and filter support the fused deposition modeling (FDM) or the droplet-based precision extrusion deposition (db-PED). These technologies, that demonstrated high resolution and reproducibility of the final product [2], avoid the use of support materials. The typical home version 3D printer [7] has also advantages, such as a low volume (cube, l = 50 cm), a reduced power consumption (nearest 330 W), and very low prices (starting from 100€ for plastic). Instead, the selected industrial model db-PED (total volume 800 × 1240 × 1885 mm) uses materials directly in pellet form with an accurate processes control to assure the repeatability of the final product. In this study, we proposed three types of mask models and, to assure recycling or reusability, we selected: food paper for the frame of the first emergency mask; polylactic acid (PLA) [28] for the 3D printed biodegradable model; advanced polyolefin (Supplementary 2) and styrene ethylbutylene styrene (SEBS) compound (Supplementary 3) for the flexible model fabricated with PED.

In detail, SEBS is a medical grade translucent compound with a hardness of 30 shore, suitable for injection molding and profile extrusion; the advanced polyolefin is specially designed for the use as a sealing or metallized layer in coextruded film applications; while PLA is one of the most used polymers in 3D printing [29]. 

The filter was made adopting the polyethylene terephthalate (PET) for the outer layer deposition and the polivinilidenfluoruro (PVDF) for the nanomembrane inner layer, developed via electrospinning [30] (dimensions 1460 × 2031 × 1435 mm). This latter technology offers a higher resolution and great advantages in material processing over a wide range of applications, such as biosensors [31], tissue engineering [32,33], drug delivery [34], and supercapacitors [35].

Finally, we evaluated the direct toxicity of the 3D printed models—in accordance with ISO 10993-5 guidelines for cytotoxicity of porous materials—and the mechanical performances before and after cleaning and disinfection, analyzing the surface roughness and nanomembrane dissolution to validate the possibility to clean/reuse the masks.

## 2. Materials and Methods

### 2.1. Mask Models

#### 2.1.1. Frames of Emergency Masks

We fabricated two models of “emergency” masks, selecting two frames: disposable and cleanable. The first frame was developed with a conventional paper pizza box (Grande D S.R.L., Vigevano (PV), Italy), mod. 32.5 DRIN (regulation CE no. 1935/2004, DPR 777/82, DM 21/03/1973). We designed the drawing to obtain a punched sheet and, by flexing the resulted product, we achieved the final shape appropriate for face adhesion. The second frame was selected from the typical silicone masks available in hospitals (Xiamen Better Silicone Rubber Co., Ltd, Xiamen, China), 22 mmID inflatable and useful to substitute the frame of the paper pizza box mask. 

#### 2.1.2. Filter Support and Eco-Masks Frame

We fabricated the filter support with two principal components: the main support, designed to collect the filtered air exploiting the whole surface of the interchangeable filter; and the grid, useful to fix the filter during breathing. Finally, it was required the production of a third part to lock the main component to the mask frame. All these parts were printed by a custom-made Delta DMake 3D printer (Futura Group SRL, Gallarate, Italy) with PLA filament bobbin (175N1, Velleman Inc., Legen Heirweg, Gavere, Belgium) without color pigments (diameter 1.75 mm (1/16′), density 1.25 g/cm^3^ (at 21.5 °C)). The printing speed was set at 15mm/s, while the hot-end and printing platform temperatures were 191 °C and 50 °C, respectively. The 3D models were designed using SolidWorks2015 (Solidsolution, London, UK), the 3D printed PLA objects were sliced with a custom-made Slic3r open-source software and the same manufacturing procedure was used for the printed mask frames.

#### 2.1.3. Self-Adaptable Mask

We designed the mask frame dividing the model into two functional parts, hard and soft, both printed using the Freeformer 200-3X (Arburg, Loßburg, Germany). The “hard” part, fabricated with Adsyl 5C30F advanced polyolefin material (LyondellBasell, Rotterdam, Netherlands) and typically used for food packaging and industrial applications, was designed to never be in contact with the skin (thus avoiding problems caused by its toxicity), and it was equipped with filter support and holes for the elastic cords. The “soft” part, fabricated with Cawiton MT930 medical-grade SEBS compound (Wittenburg BV, Zeewolde, The Netherlands), was equipped with an internal membrane that gives the possibility to self-adapt the mask shape (age, gender, etc.), thus improving the contact surface and reducing stress (generated by the elastic cords). The printing speed was 20 mm/s for the contour and 110 mm/s for the infill, the hot-ends temperature was fixed between 210–220 °C, while the environment one at 80 °C; the production time was about 32 h.

### 2.2. Cleaning and Disinfection

To reuse the presented masks, we evaluated different types of cleaning/disinfection methods [36,37]. Following the ISO 527, we printed 15 samples for each selected material (PLA, polyolefin, SEBS), and only 10 of them were cleaned in a typical water solution used for clothes cleaning (10 g/l detergent SOLE Reckitt Benckiser, Milan, Italy). The solution was stored with the samples in a climatic chamber (Angelantoni DY 110, Massamartana, Italy) for 4 h at 60 °C and 90% of humidity, and subsequently dried for 1 h at 60 °C and 10% of humidity. Furthermore, 5 of the previous 10 cleaned samples, for each material, were disinfected with an EtOH 95% solution. In summary, we printed 45 samples (15xPLA, 15xPolyolefin, 15xSEBS), implementing statistical analyses on the 5 samples for each cleaning phase (5xNot treated, 5xCleaned, 5xCleaned and disinfected).

### 2.3. Material Mechanical Characterization

The mechanical characterization analyses were performed using MaCh5 (MaCh3D SRL, Parma, Italy), a commercial desktop testing machine (load capacity of 5kN and a crosshead run of 110 mm) [38]. The specimen free trait was considered as base length for strain measurements and, according to ISO 527, it was set to a length of 60 mm between rollers’ contact lines and specimens end-tabs.

The testing machine was equipped with special grips (Figure S1), the test speed was set to 5 mm/min, the sample rate was 5Hz, and we evaluated, as previously described, 45 specimens.

### 2.4. Morphological Characterization

The morphological surfaces of the sample materials (PLA, polyolefin, SEBS) were analyzed before (just printed mask) and after cleaning/disinfection (materials processed and ready to be used) by acquiring the SEM images (Carl-Zeiss Auriga Compact SEM-FIB, Oberkochen, Germany) obtained from the specimens used for the mechanical characterization. First, we cut 5 × 4 mm pieces of 3D printed polymers, using an Edwards Scancoat-six sputter with 1 kV voltage and 30 mA current for 2 min, and then we sputtered a thin gold layer (10 nm) on the surface of the samples to guarantee the conductive back contact. Successively, samples were signed and lodged into the SEM chamber radial stub.

Each sample was fixed through a conductive carbon tape in order to guarantee both the stability of the sample on the stub and the back-conductivity. Afterward, we acquired pictures from at least 3 different points of the same surface area to ensure the reliability of the analyzed morphology.

### 2.5. In Vitro Biological Tests

All the cleaned+dried+disinfected, cleaned+dried samples and not cleaned, control samples (treated with UVC light for 20 min before cell seeding to prevent the undesired spread of microorganisms, necessary condition for cell culturing) were tested for cell viability and metabolic activity after 24 h growth (following the ISO 10993-5 guidelines for the toxicity study of porous materials) [39], evaluating the direct effect of PLA, polyolefin and SEBS materials on A431 human epidermal cells. A431 human epidermal cells were seeded onto each group of samples at a final density of 1 × 10^4^ cells/sample and cultured at 37 °C in D-MEM (Thermo Fisher Scientific, Carlsbad, CA, USA) supplemented with 10% Fœtal Bovine Serum (FBS, Thermo Fisher Scientific) and 1% antibiotics (Penstrep, Thermo Fisher Scientific). A control group was characterized by cell growth on TCPs to normalize experimental results.

After a 24 h incubation, the cell proliferation was quantified with chemiluminescence assay (CellTiter-GLO, Promega, Madinson, WI, USA), following the manufacturer’s recommendations. Briefly, the culturing medium was removed; the samples were rinsed in Phosphate Buffer Saline (PBS, Thermo Fisher Scientific) and incubated with a 50:50 solution of lysis buffer and fresh medium. The specimens were shacked for 2 min and the developed luminescence was stabilized for 10 min at room temperature in the dark. Samples luminescence was finally assessed with a GLOMAX 20/20 luminometer (Promega). An MTT test (Sigma-Aldrich, Saint-Louis, CA, USA) was developed to quantify the cellular metabolic activity: the culturing medium was removed and the samples were incubated for 4 h at 37 °C with 1:10 kit-provided labeling reagent-medium solution. After the incubation time, the solubilization solution was added and let overnight at 37 °C in the dark. The samples absorbance was then quantified through a measured Multiskan FC plate reader (Thermo Fisher Scientific) at 620 nm.

### 2.6. Filter and Melting Analyses

The filter membrane, fabricated with hybrid electrospinning [30] technology (NanoSpinner PilotLine-PE550, Inovenso, Istanbul, Turkey), was composed of three layers: the top and the bottom were made of PET Spunbond 35 g/m^2^, and the internal nanofiber layer was printed with polymer PVDF 0.6/0.8 g/m^2^ (Inovenso, Istanbul, Turkey).

The filter reached the required 99% of bacterial filtration efficiency (Supplementary 4 and 5), respecting all the directives defined from the EN 143 for the P3 medical device marking (Supplementary 6), and it was kindly donated by Vivida (Copenhagen, Denmark), which supported the experimental tests.

The filter, immersed for one hour at 50 °C in distilled water and subsequently dried for one hour at 50 °C with 10% of humidity in the climatic chamber (Angelantoni DY 110, Massamartana, Italy), was analyzed with SEM technology, following the same method utilized for the surface evaluation of the extruded materials (mask frame).

### 2.7. Statistical Analysis

The data obtained are reported as the mean ± SD and were analyzed using Prism7 (GraphPad, La Jolla, CA, USA). Differences were considered significant when *p* < 0.05 and evaluated with a two-way ANOVA statistical test or Tukey multiple comparison post-hoc test.

## 3. Results and Discussion

### 3.1. Prototyped Masks

**Figure 1:**
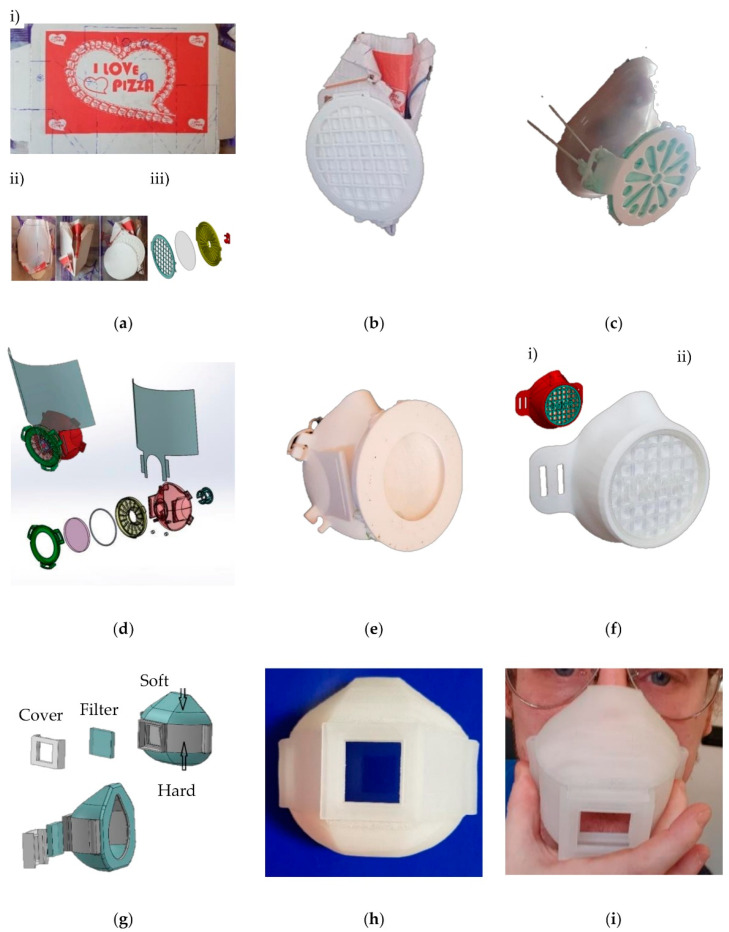
Prototyped masks: (**a**) Schematics of emergency masks, (**i**) shape design, (**ii**) details of mask, (**iii**) details of filter support; (**b**) Details of paper mask and 3D printed PLA filter support; (**c**) Filter support connected with a typical hospital silicone mask; (**d**) Schematics of eco-masks for hospital use; (**e**) Detail of hospital eco-mask with removable support filter; (**f**) eco-mask for industrial use with lock-grid for filter change: (**i**) model design, (**ii**) prototyped mask; (**g**) Schematics of self-adaptable masks fabricated with droplet-based precision extrusion deposition (db-PED); (**h**) Detail of resting mask shape; (**i**) Final shape of the universal self-adaptable mask, obtained with PED technology.

Figure 1a shows the first prototyped mask, a user-friendly model, able to satisfy the high number of requests of SPD. 

In particular, to reduce the production time, the frame was fabricated with a paper pizza box (Figure 1a(i)) for its thermal resistance, non-toxicity, recyclability and ability to absorb sweat, and it was designed considering the absence of cutting edges and holes (Figure 1a(ii)), the ergonomics and the shape customization. 

The filter support (Figure 1a(iii)) with air collectors was created through technologies and materials available at home: non-toxic and biodegradable PLA, which is the easiest 3D printable material available in the form of bobbins; and low-costs 3D printers based on FDM additive manufacturing technology without environmental temperature control.

For the fast filter locking, the filter support was connected with a plug-grid that gives the possibility to change the filter easily, reducing the problem of its saturation and contamination, very useful in surgery safety maintenance.

The time required to produce the filter support (Figure 1b) was 3 h and 40 min, adding some minutes for the mask frame modeling. 

Unfortunately, we cannot use this type of “emergency mask” (Figure 1b) in the hospital context, because it was not able to assure safety in long-term uses, for instance, humidity can damage the paper. Moreover, recent studies have demonstrated that paper pizza box (general food packaging material) can be dangerous for the COVID vaccine treatments [40]. 

Thus, we adopted the already present silicone masks used in hospitals, modifying the plug-grid design for the elastic cord connection (Figure 1c) and the filter support for the fast filter change. This model required 5 h and 38 min of manufacturing time. However, the standard silicone mask guaranteed face adherence only adding tension force on the elastic cords that, however, reduces the time of portability.

For these reasons, we printed a new type of mask fully biodegradable. This new model keeps the same approach applied for the fabrication of the filter and the related support. In addition, we included in the design of the frame the human face morphology and some gadgets, like the splash shield support and the one-way valves (Figure 1d), improving the portability for long surgery activities. The presented model (Figure 1e) maintained also the possibility to connect hospital ventilators like the silicone mask model, but it requires a high 3D printing platform surface and development time (20 h and 37 min) to obtain all the components in a single printing procedure. Thus, to satisfy the necessity in industrial areas (reduced fabrication time, reduced gadgets requirements, high numbers of ergonomic masks), we customized this model (Figure 1f) merging the filter support and the mask frame in one block and adding a custom plug-grid for the person identification during working activity (Figure 1f, blue cap “UNIPR”). The 3D printer requires 11 h and 59 min to produce this type of mask.

The last “self-adaptable” mask model (Figure 1g) was implemented using two food-grade materials: advanced polyolefin for the structural maintenance and filter adaptation; SEBS compound for the flexibility (Figure 1h,i). This characteristic of SEBS facilitates self-adaptation without the necessity to scan the user face to customize the morphology, thus reducing the fabrication time that overcomes 24 h. These materials, thanks to the db-PED technology, can be used starting directly from the polymers pellet form and avoiding the processes and issues related to the fabrication and employment of the bobbin, typically used in the FDM technology. Moreover, PED technology enables the possibility to functionalize polymers and composite materials adding, for instance, silver nanoparticles in the pellet fabrication (improving safety and self-cleaning functions in the final 3D printed product).

### 3.2. Mechanical Characterization

**Figure 2:**
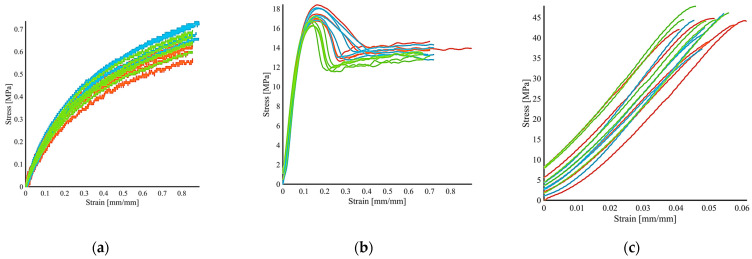
Stress-Strain curves of tested material: (**a**) SEBS; (**b**) polyolefin; (**c**) PLA. Not treated, green lines; Cleaned, blue lines; Cleaned and dried, red lines.

To assure the global scaling (ability to be worn by children and adults), reuse and ergonomics of the model, we studied mechanical characteristics (Figure 2, Table S1), surface morphology, toxicity and membrane dissolution on the dermal cells of the 3D printed polymers, before and after the cleaning/disinfection activity.

It is possible to appreciate the different mechanical behaviors of the tested materials (Figure 2). The SEBS material (Figure 2a) showed high deformability and the ability to maintain the initial dimensions after testing typical behavior for elastomeric materials, such as rubbers, which present elongations up to 500% before failure. Setting the crosshead run to 60mm, we did not break the specimens reaching strain levels up to 80% without showing any failure sign. On the other hand, advanced polyolefin material (Figure 2b) presents a stress peak, and specimens can withstand strains in the order of 70% before failure, showing an intermediate behavior in terms of elongation at break and ultimate stress between SEBS and PLA. At last, PLA material (Figure 2c) presents a more fragile nature, denoting higher rigidity compared to the previous ones as well as mechanical strength. Detailed results can be found in Table S1: average elastic modulus (E) for SEBS is 2.33 ± 0.11 MPa and ultimate stress (R_m_) 0.66 ± 0.05 MPa, for polyolefin E = 191.7 ± 12.4 MPa, R_m_ = 17.13 ± 0.47 MPa and for PLA E = 896.9 ± 26.66 MPa, R_m_ = 43.40 ± 1.74 MPa. Moreover, PLA (porous and hydrophobic) and SEBS (hydrosoluble) materials demonstrated the ability to be cleaned at least once, regaining the starting material properties after the drying phase, justifying the manufacturing time required for the shape customization or industrialization. In this case, rupture happens in the range of 4–6% deformation.

From the graphs, it is not evident an influence of different treatments. The adoption of this material for long-term uses will require mandatory specific experimental tests. With the aim to achieve the same result in terms of 3D object performance and safety during the final use, we will, for instance, increase the wetting time of specimens storing and assure fine parameter control of the additive manufacturing parameters (environmental, speeds, etc.).

**Figure 3:**
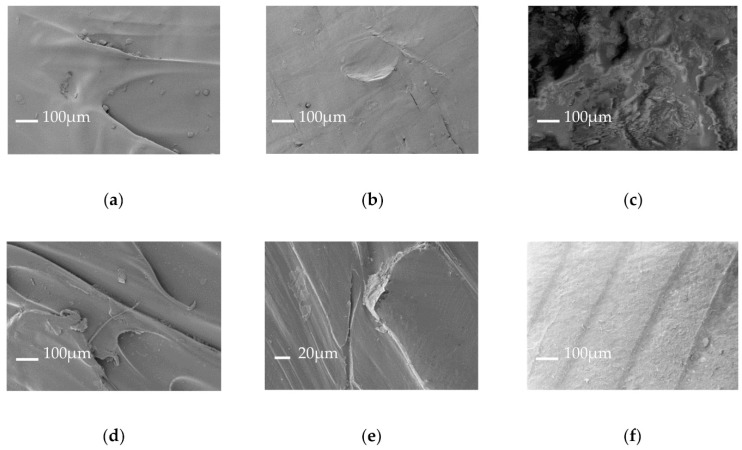
SEM analysis of 3D printed polymer surfaces: (**a**) Detail of just printed PLA; (**b**) Detail of just printed polyolefin polymers; (**c**) Detail of just printed medical-grade SEBS polymer; (**d**–**f**) Printed surface after cleaning/disinfection of printed polymers used for the mask realization: PLA, polyolefin and SEBS respectively.

The morphological surface of all the samples was analyzed by acquiring SEM images before and after the cleaning and disinfection phases (Figure 3).

Before cleaning, we noticed that PLA and polyolefin show a surface with small ripples (Figure 3a,b), which increase after cleaning/disinfection (Figure 3d,e); while, before cleaning, the SEBS surface is very difficult to detect (Figure 3c), but the cleaning/disinfection phase improves its uniformity at a higher resolution (Figure 3f). Thus, we have to compare the particle dimensions with the typical fiber, released from disposable masks, and eventually to add an internal filter, able to lock this micro-waste.

### 3.3. In Vitro Biological Tests

**Figure 4:**
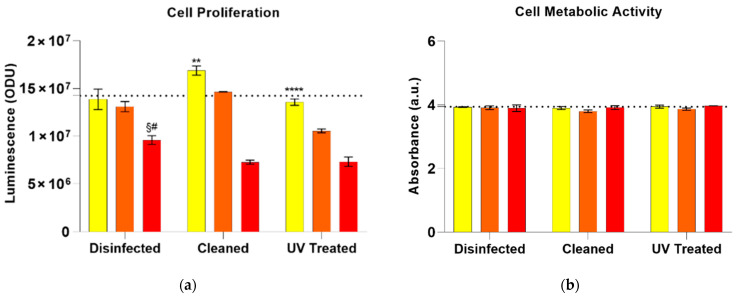
(**a**) Cell proliferation assay (**b**) and metabolic activity test on A431 epithelial cells after 24 h of culture. ** *p* = 0.012 PLA cleaned vs. SEBS cleaned; **** *p* < 0.0001 PLA UV treated vs. SEBS UV treated; § *p* = 0.0008 polyolefin disinfected VS. polyolefin cleaned; # *p* = 0.001 polyolefin disinfected VS. polyolefin UV treated. Dotted lines = TCPs control value. Yellow: PLA; Orange: SEBS; Red: polyolefin.

We performed a direct contact cytotoxicity test, in agreement with ISO 10993-5 guidelines, to observe the effect of direct contact between cultured A431 human epidermal cells and the PLA, polyolefin and SESB substrates. Chemiluminescence, a quantitative test measuring ATP content and cell proliferation, showed a great response of cells in regard to all the tested samples (Figure 4a) after 24 h of direct contact with the materials. This is fundamental because the use of such a mask provides a medium duration time of 4–6 h, much lower than the tested one.

Not surprisingly, the assays underlined a slower proliferation rate on all the polyolefin samples ((9.6 ± 0.46) × 10^6^ and (7.2 ± 0.22) × 10^6^ for disinfected and cleaned respectively), probably due to the chemical composition of polyolefin (typically used for the development of the support structure, never in direct contact with the skin).

Polyolefin data are clearly in contrast with the PLA and SEBS results, where the assays underlined a much higher cellular proliferation: (13.8 ± 1) × 10^6^ and (13,10.57) × 10^6^ on disinfected PLA and SEBS respectively, (16 ± 0.48) × 10^6^ on the cleaned PLA and (14 ± 0.027) × 10^6^ on the cleaned SEBS.

PLA, as the positive control, showed a high proliferation rate even if compared to TCPs samples (Figure 4a—dotted line), underlining the non-cytotoxic effect of the substrate. Interestingly, the SEBS disinfected samples presented no significant differences if compared to PLA samples, while in cleaned and UV-treated control groups the proliferation value was slightly lower (PLA cleaned vs. SEBS cleaned *p* = 0.012 and PLA UV-treated vs. SEBS UV-treated *p* < 0.0001). The cellular proliferation on polyolefin surfaces was lower on all the tested samples, nevertheless, as expected, the disinfected group presented a higher number of cells at 24 h (polyolefin disinfected vs. polyolefin cleaned *p* = 0.0008 and polyolefin disinfected VS. polyolefin UV-treated *p* = 0.001).

MTT results (Figure 4b) showed that cell metabolic activity was completely unaffected by both, the analyzed substrates and the cleaning methods, highlighting a cellular viability as high as on the control samples (TCPs—Figure 3b dotted line), presenting medium values very comparable (PLA: disinfected 3.93 ± 0.01, cleaned 3.91 ± 0.04, UV-treated 3.94 ± 0.05; SEBS: disinfected 3.9 ± 0.05, cleaned 3.8 ± 0.04, UV-treated 3.86 ± 0.04; PP: disinfected 3.89 ± 0.10, cleaned 3.91 ± 0.06, UV-treated 3.97 ± 0.001).

The preliminary results from the cell culture study (Figure 4) confirmed that all the three selected materials can be used in direct contact with the skin, preferentially after preparation through disinfection or normal cleaning. Further in vitro assays have to be performed to investigate the cytocompatibility of the biomaterials for longer times and the release of possible cytotoxic agents in the cells culture medium.

### 3.4. Electrospinned Filter

**Figure 5:**
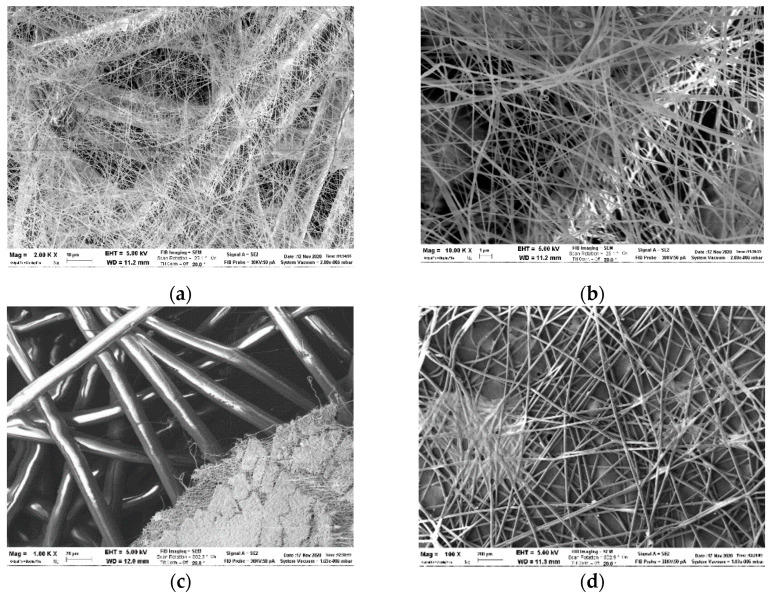
Electrospinned filter: (**a**) Detail of electrospinned membrane before cleaning and disinfection; (**b**) Detail of filter after cleaning and disinfection; (**c**) Detail of microfiber (i) and nanofiber (ii) after cleaning and disinfection; (**d**) Detail blend-like area of melted nanofiber membrane between the nanofiber and microfiber layers.

In our study, the electrospinned filter is equipped with a microfiber membrane covered by a water-soluble nanofiber membrane. Figure 5 shows the nanomembrane dissolution SEM images of the electrospinned fiber samples before and after cleaning-disinfection treatment. More in detail, immersing the nanofiber for 1h at 50 °C, there is a formation of a blend-like area between the two layers on the microfiber support membrane.

Figure 5a,b show the membrane before (5a) and after (5b) cleaning/disinfection at higher magnification, showing details of the texture. Figure 5c highlighted the different behavior of the microfiber and nanofiber after dissolution, the PET fiber remains unaltered, while the PVDF membrane changes completely its morphology. In Figure 5d, it is possible to appreciate the effect of the nanofiber dissolution and adhesion directly on the microfiber.

Changing its morphology, the nanofiber membrane assures the starting permeability, covering the holes and reducing breathability. Thus, we can assume that it can be used like an index performance, related to the aging of the filter: when we exceed the suggested hours of usage, breath becomes difficult because its humidity dissolves the nanomembrane by the time, causing the filter saturation. This happens also with powder filters, where particles tap every holes.

## 4. Conclusions

The realization and utilization of 3D printed masks require the adoption of cleaning and disinfection procedures for the different available materials and the related applications. In the present study, these processes modified the PLA and SEBS substrate morphology, demonstrating the possibility to reduce the surface roughness and the related risk of bacteria and viruses’ accumulation.

Moreover, while the rigid PLA requires shape customization for an ergonomic, long-time use without skin stress, we can fabricate flexible and adaptable masks using, for instance, polyolefin and SEBS with 3D equipment, allowing the finer environmental temperature control and the use of pellet polymers.

Finally, comparing the mechanical, morphological and viability tests of the PLA home-printed mask models, the results demonstrated similar performances to the other prototyped objects. We can also assume that it is possible to use home-made 3D printed masks at least once before recycling them, without a finer standardization of the manufacturing temperature. Then, we can use typical home materials for the cleaning and disinfection procedures while avoiding toxic adhesive platform materials like pigments inside PLA.

Industrial 3D printers give the possibility to use specialized materials, also with food contact certification, and to reduce time as well as the potential contamination from bobbins. Moreover, the isolated printing environment enables the fast filtration of air and pollution generated during fabrication. Moreover, the 3D printer should be included inside a clean room, mandatory to validate and certify materials/technologies/designed model for each 3D printed product and related final use.

This study aims to support scientists and designers in the development of new research approaches to the additive manufacturing post-processing phases, indispensable to assure human safety in the fabrication of 3D printed custom medical devices.

## Supplementary Materials

The following are available online at https://www.mdpi.com/2073-4360/13/4/617/s1, Figure S1. MaCh 5 Universal Testing Machine with roller grips, Table S1: mechanical performance of the 3D printed polymers before and after cleaning/disinfection treatment. Supplementary 2. Polyolefin datasheet. Supplementary 3. SEBS datasheet. Supplementary 4. Bacterial Filtration Efficiency and Differential Pressure Final Report. Supplementary 5. Filtration efficiency. Supplementary 6. Respiratory protective devices-Particle filters-requirements, testing marking.
